# Inverted Rearfoot posture in subjects with coexisting patellofemoral osteoarthritis in medial knee osteoarthritis: an exploratory study

**DOI:** 10.1186/s13047-018-0261-6

**Published:** 2018-05-08

**Authors:** Hirotaka Iijima, Hiroshi Ohi, Naoto Fukutani, Tomoki Aoyama, Eishi Kaneda, Kaoru Abe, Masaki Takahashi, Shuichi Matsuda

**Affiliations:** 1 Department of System Design Engineering, Keio University, Yokohama, Japan; 2 Department of Physical Therapy Human Health Sciences, Graduate School of Medicine, Kyoto University, Kyoto, Japan; 3 Japan Society for the Promotion of Science, Tokyo, Japan; 4 Graduate School of Health and Welfare, Niigata University of Health and Welfare, Niigata, Japan; 5 Ohi Manufacturing Co., Ltd., Kyoto, Japan; 6 Nozomi Orthopedic Clinic, Hiroshima, Japan; 7 Department of Orthopedic Surgery, Graduate School of Medicine, Kyoto University, Kyoto, Japan

**Keywords:** Rearfoot, Patellofemoral osteoarthritis, Inversion

## Abstract

**Background:**

While abnormal rearfoot posture and its relationship to patellofemoral (PF) pain has been thoroughly discussed in the literature, its relationship to patellofemoral osteoarthritis (PFOA) has not been determined. This study aimed to examine whether rearfoot posture is associated with a higher prevalence of radiographic PFOA in a compartment-specific manner in patients with medial tibiofemoral osteoarthritis (TFOA).

**Methods:**

Participants from orthopedic clinics (*n* = 68, age 56–90 years, 75.0% female), diagnosed with radiographic medial TFOA (Kellgren/Lawrence [K/L] grade ≥ 2) were included in this study. The presence of PFOA and static rearfoot posture were evaluated using a radiographic skyline view and a footprint automatic measurement apparatus, respectively. The relationship between rearfoot posture and PFOA was examined using analysis of covariance and propensity score-adjusted logistic regression analysis.

**Results:**

On average, patients with coexisting PFOA and medial TFOA (*n* = 39) had an inverted calcaneus 3.1° greater than those with isolated medial TFOA (*n* = 29). Increased calcaneus inverted angle was significantly associated with a higher probability of the presence of medial PFOA (odds ratio: 1.180, 95% confidence interval: [1.005, 1.439]; *p* = 0.043). Calcaneus inverted angle was not associated with higher odds of lateral PFOA presence based on the adjusted values.

**Conclusions:**

The presence of an inverted rearfoot was associated with PFOA. Although these findings do not clearly indicate a biomechanical link between rearfoot posture and PFOA, this study shed light on the potential relationship between altered rearfoot posture and PFOA, as can be seen between rearfoot abnormality and PF pain.

## Background

Knee osteoarthritis (OA) is the leading cause of knee pain and disability worldwide [1]. Patellofemoral (PF) osteoarthritis (PFOA) is an under-recognized, yet important, subgroup of knee OA [2, 3]. Depending on the source population and definition of OA, PFOA is present in 32–57% of adults [4] and commonly occurs in combination with tibiofemoral (TF) OA (TFOA) [5–7]. The risk factors in OA pathogenesis vary according to the affected compartment [8–10] and targeted interventions for PF joint disease are required owing to the unique biomechanics of the PF joint [2, 11]. However, the factors that contribute to its development and the effective management of this common and potentially debilitating condition [3] have not been elucidated.

The rearfoot affects the biomechanical alignment of the lower limb [12, 13], and therefore, it has the potential to lead to proximal diseases including PFOA [14]. Tibeiro et al. hypothesized that excessive eversion of the rearfoot can lead to increased tibial and femoral internal rotation, subsequently resulting in higher lateral PF cartilage stress due to increased knee valgus and quadriceps angle [15], although this has not proven yet. The relationship between altered foot posture during gait and PF pain was thoroughly discussed in the literature [16] and rearfoot abnormalities may be associated with PF pain [17]. The clinical symptoms and functional limitations between PFOA and PF pain in adolescents and young adults [2, 18] are similar, and approximately 70% of cases have radiographic PFOA in subjects (aged > 40 years) with PF pain [5]. Therefore, it is possible that an altered rearfoot posture would be modifiable factors associated with PFOA, as can be seen between rearfoot posture and PF pain. Although foot orthoses were effective in improving anterior knee pain in patients with isolated lateral PFOA [19], we are not aware of any studies that investigated the relationship between rearfoot posture and PFOA.

Static rearfoot posture can be easily evaluated in the clinical setting without sophisticated equipment and has been used in assessments of patients with PF pain [20]. Investigating rearfoot posture in patients with coexisting PFOA may assist clinicians to better understand the effect of rearfoot posture on PF pathology and may add to the limited evidence of studies with PFOA population. Thus, this study aimed to examine whether altered static rearfoot posture is associated with higher prevalence of radiographic PFOA in patients with medial TFOA in a compartment-specific manner. Such a relationship would indicate the existence of a biomechanical association, necessitating a prospective cohort study to find modifiable risk factors for the incidence and progression of PFOA.

## Methods

### Participants

Participants of this exploratory study were recruited from the 12-month follow-up period of a prospective cohort of subjects described in a previous study, which investigated the clinical impact of coexisting PFOA in patients with medial TFOA [21]. Briefly, 143 patients with medial knee OA were recruited from a community orthopedic clinic in February 2014, and were followed up for 12 months. The patients, diagnosed by their attending physician, were recruited through advertisements and followed up for 12 months. The inclusion criteria were (i) age ≥ 50 years; (ii) radiographic OA (i.e. Kellgren/Lawrence [K/L] [22] grade ≥ 2) primarily in the medial TF compartment in one or both knees, as evaluated by weight-bearing anteroposterior radiographs; and (iii) the ability to walk independently on a flat surface without any ambulatory assistive device. Subjects at baseline were included if they had medial TFOA, regardless of PFOA status. No restriction was imposed on laterality; both patients with bilateral and unilateral radiographic knee OA were included in this study. The exclusion criteria were: (i) a history of knee surgery, (ii) inflammatory arthritis, (iii) periarticular fracture, (iv) current neurological problems, or (v) lateral TFOA. Lateral TFOA was defined as a knee having a K/L grade ≥ 1 along with joint space narrowing (JSN) > 0 in the lateral compartment with JSN = 0 in the medial compartment [23]. In other words, only patients who had a more severe radiographic disease in the medial compartment compared to the lateral compartment (i.e., isolated medial TFOA or mixed medial and lateral TFOA) were included in this study. Since medial and lateral knee OA have distinct characteristics [24], and most knee OA is the medial type in Japan [25, 26], lateral TFOA (i.e., lateral OA severity > medial OA severity) was excluded in this study. The Ethical Committee of Kyoto University approved this study (approval number: E1923). Written informed consent was obtained from all participants at baseline and at 12 months follow-up.

### Radiographic PF joint disease severity

The radiographic data of the lateral and skyline views at baseline were obtained from all participants. If clinical symptoms worsened within the 12-month follow-up period, participants underwent repeat radiography. Detailed methods of radiographic evaluation of disease severity in the PF joints were described elsewhere [21]. Briefly, a single trained examiner (HI) assessed radiographic severity for the PF joint using the K/L grading system adapted to the lateral and medial facets of the PF joint. Presence of PFOA was defined as knee with K/L grade 2 in skyline view or osteophytes 1 in lateral view. We have previously reported excellent intra-rater reliability for such radiographic evaluation (Kappa: 0.80) [21].

### Static Rearfoot posture

Static foot posture was evaluated in January 2015 using a three-dimensional automatic footprint measurement apparatus (CUTE, JMS-2100CU; Dream GP Inc., Osaka, Japan) [27, 28]. This foot scanning system is based on laser line triangulation, where the measuring head moves around a single foot in an oval-shaped trajectory [29]. The laser scanner rotates around the patient’s foot and measures more than 30,000 points, including the ankle, instep, heel, toes, as well as the sole, thereby precisely re-creating the patient’s foot shape. This scanning system has a high accuracy for measuring foot posture. The measurement errors of foot length and foot width are − 0.27-0.36 mm (accuracy ±0.2%) and 0.51–1.22 mm (accuracy ±0.5%), respectively [29].

**Fig. 1:**
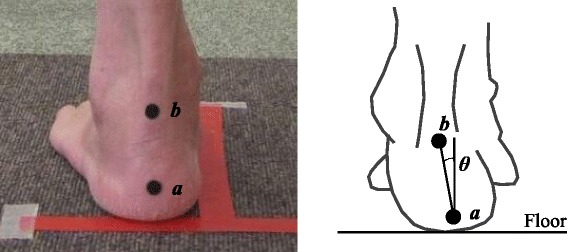
Measurement of calcaneus inverted angle relative to the floor (θ). Calcaneus inverted angle relative to the floor was automatically calculated by the system according to the attached round black seal markers (*a* bottom of the calcaneal tuberosity; *b* enthesis of the Achilles tendon)

Prior to each capture session, the patient was asked to stand on bare feet with shoulder-width apart. This allowed 50% of their body weight to be placed on each foot during assessment. Round, black seal markers, which corresponded to 2 anatomical landmarks (i.e., bottom of the calcaneal tuberosity and enthesis of the Achilles tendon) to detect foot alignment, were attached to the skin (Fig. 1). After the measurements, foot length and calcaneus inverted angle relative to the floor were automatically calculated by the system according to the attached round black seal markers. As a clinical frame of reference, calcaneus inverted angle relative to the floor were categorized based on value of foot posture index subcategory “inversion/eversion of the calcaneus” [30] as follows: everted calcaneus (calcaneus angle ≤ − 5°); normal calcaneus (− 5° < and ≤ 5°); and inverted calcaneus (calcaneus angle > 5°). Throughout the manuscript, “inversion/eversion” indicates posture on a single frontal plane, which is a part of triplane motion “supination/pronation”.

### Covariates

Data on age, sex, and height were self-reported by patients. Patients wearing clothes without shoes were weighed on a scale. Body mass index (BMI) was calculated by dividing the weight by height squared. Radiographic medial TFOA severity and anatomical axis angle (AAA) with sex-specific correction [31] were assessed in the anteroposterior short view in the weight-bearing position. The intra-rater reliability was excellent for evaluating the TFOA K/L grade (Kappa: 0.80) and measuring the AAA (intra-class correlation coefficient [ICC]: 0.98). Varus thrust was evaluated according to previously described methods [32, 33]. Two physical therapists judged the presence of lateral movement of the tibial tuberosity relative to hip and ankle. This resulted in an increase in varus alignment during initial contact with the mid-stance of the stance phase with self-selected speed. We have previously reported good interrater reliability (Kappa: 0.73) for evaluating varus thrust assessment [32, 33]. A trained physical therapist passively measured the flexion and extension range of motion (ROM) of the affected knee joint through standard goniometric procedures according to previously validated methods [34].

### Statistical analyses

To minimize any bias produced by similarities between the knees of the same patient, only one knee per patient was analyzed, which was designated as the “index knee.” The index knee was defined as the more painful knee in the present or past. Descriptive statistics were calculated as mean and standard deviation (SD) for continuous variables and as proportion for dichotomous/categorical variables. We performed univariate analysis using Student’s *t*-test for parametric continuous variables and Fisher’s exact test for dichotomous/categorical variables and compared the differences in rearfoot posture between knees with and without PFOA. Subsequently, the values of calcaneus inverted angle were compared using analysis of covariance. Covariates included age, sex, BMI, TF joint K/L grade, corrected AAA, presence of varus thrust, and knee flexion ROM. These covariates were chosen based on clinical judgment and previous studies investigating factors associated with PFOA or rearfoot posture [21, 35–37]. The normality of calcaneus inverted angle was assessed using the Shapiro-Wilk test and the homogeneity of the variances between patients with and without PFOA was confirmed using the F-test.

We further performed multiple logistic regression analyses and calculated odds ratios (ORs) and their 95% CIs. Logistic regression analyses were performed first with an unadjusted model and then with a propensity adjusted model. Due to the small sample size, we used propensity score adjustment including the above covariates. Data analyses were performed with JMP Pro 12.2 (SAS Institute, Cary, NC, USA). *P*-values < 0.05 were considered statistically significant.

## Results

**Fig. 2:**
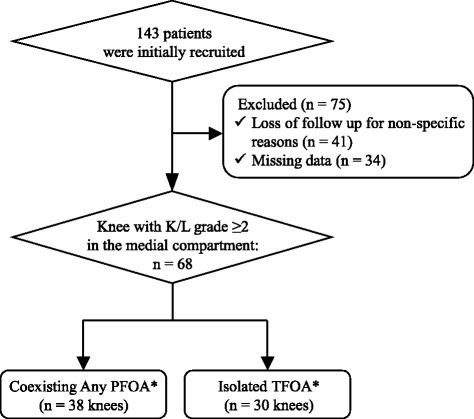
Flowchart showing the inclusion of participants in the study. *Presence of PFOA was defined as knee with K/L grade 2 in skyline view or osteophytes 1 in lateral view [21] based on baseline radiography. If clinical symptoms worsened within the 12-month follow-up, participants underwent repeat radiography that is used for evaluation of the PFOA presence

Figure 2 shows flowchart describing the inclusion of study participants. One-hundred-forty-three patients with medial knee OA were enrolled at baseline period (February 2014). Of these patients, 75 were excluded from this study; 41 could not be contacted or declined follow up for non-specific reasons; and 34 were excluded because of missing data on patient’s characteristics, radiography, and rearfoot posture at the 12 months follow up period (January 2015). Thus, our final sample included 68 patients (47.6% of the initial cohort) at 12 months follow-up period. Baseline characteristics were compared between included and excluded patients, and no significant differences were found between the two groups in terms of demographic characteristics and radiographic disease severity at baseline period (data not shown). Of the 68 patients who completed the study (age 56–90 years; 75.0% female), 48 (70.6%) had mild diseases with K/L grade = 2 in their index knee (Table 1). Thirty-eight (55.9%) of these patients had PFOA.

**Table 1:** Patients’ characteristics at follow up period (*n* = 68)^a^

Age, years	74.69 ± 7.785
Female, no. (%)	51 (75.0)
Body mass index, kg/m^2^	24.14 ± 3.753
Corrected anatomical axis angle, degrees	176.2 ± 4.951
Presence of varus alignment, no. (%)^a^	55 (80.9)
Medial tibiofemoral joint K/L grade, no. (%)^b^	
Grade 2	48 (70.6)
Grade 3	11 (16.2)
Grade 4	9 (13.2)
Presence of varus thrust, no. (%)	12 (17.6)
Knee range of motion, degrees	
Extension^c^	−6.471 ± 6.482
Flexion	140.8 ± 12.17
Presence of any PFOA, no. (%)	38 (55.9)
Presence of mixed PFOA, no. (%)	19 (27.9)
Presence of medial PFOA, no. (%)	22 (68.8)
Presence of lateral PFOA, no. (%)	26 (32.4)
Coexisting medial TFOA and any PFOA, no. (%)	38 (55.9)
Calcaneus inverted angle, degrees^d^	−0.357 ± 5.522

K/L grade: Kellgren/Lawrence grade; PFOA: patellofemoral osteoarthritis; TFOA: tibiofemoral osteoarthritis

Except where otherwise indicated, values are mean ± SD

^a^Varus alignment is defined as corrected anatomical axis angle < 179 degrees

^b^If participants did not get worse their clinical symptoms within the 12-month follow-up period, radiography at baseline was used for K/L grade assessment

^c^A negative value indicates that the knee is flexed

^d^A positive value indicates inversion direction of the calcaneus

Table 2 shows the comparison of calcaneus inverted angle and calcaneus alignment in knees with and without PFOA. Calcaneus inverted angle in patients with coexisting PFOA was higher than those with isolated TFOA (1.046 ± 5.053 vs. -2.245 ± 5.648; *p* = 0.014). Patients with coexisting PFOA on average had an inverted calcaneus 3.1° greater than those with isolated medial TFOA after adjusting for age, sex, BMI, TF joint K/L grade, corrected AAA, presence of varus thrust, and knee flexion ROM (*p* = 0.047). Patients with coexisting PFOA had a higher prevalence of inverted calcaneus (15.4% vs. 6.9%) and lower prevalence of everted calcaneus (12.8% vs. 27.6%), although these calcaneus alignments did not significantly differ between the two groups (*p* = 0.218).

**Table 2:** Comparison of calcaneus inverted angle and calcaneus alignment in knees with and without PFOA (*n* = 68)

Variables	Coexisting Any PFOA (*n* = 38 knees)	Isolated TFOA (*n* = 30 knees)	*p*-value^†^	Difference in mean (95% CI)^††^	*p*-value
Calcaneus inverted angle, degrees§	**1.046 ± 5.053**	**−2.245 ± 5.648**	**0.014**	**3.109 (0.037, 6.181)**	**0.047**
Calcaneus alignment, no (%)§§			0.218		
Everted calcaneus	5 (13.2)	8 (26.7)			
Normal calcaneus	27 (71.1)	20 (66.7)			
Inverted calcaneus	6 (15.8)	2 (6.7)			

PFOA: patellofemoral osteoarthritis; TFOA: tibiofemoral osteoarthritis; 95% CI: 95% confidence interval

Except where otherwise indicated, values are mean ± SD

^†^Based on Student *t*-test (calcaneus inverted angle) and the Fisher’s exact tests (calcaneus type) between two groups

^††^Adjusted for age, (continuous), sex (0: male, 1: female), body mass index (continuous), tibiofemoral joint Kellgren/Lawrence grade (continuous), corrected anatomical axis angle (continuous), presence of varus thrust (0: absence, 1: presence), and knee flexion range of motion (continuous)

^§^A positive value indicates inversion direction of the calcaneus.

^§§^Inverted calcaneus: calcaneus angle ≤ − 5 degree; normal calcaneus: −5 degree < and ≤ 5 degree; everted calcaneus: calcaneus angle > 5 degree.

Bold type represents a statistically significant result

Logistic regression analyses (Table 3) revealed that rearfoot posture was associated with PFOA in a non-compartment specific manner. Calcaneus inverted angle was significantly associated with higher odds of the presence of any (OR = 1.134, 95% CI [1.013, 1.291], *p* = 0.028) and medial PFOA (OR = 1.180, 95% CI [1.005, 1.439], *p* = 0.043); however, significant relationships were not confirmed mixed (OR = 1.135, 95% CI [0.958, 1.406]; *p* = 0.147) and lateral PFOA (OR = 1.078, 95% CI [0.965, 1.213], *p* = 0.183).

**Table 3:** Results of binary logistic regression analysis of the association between calcaneus inverted angle and the presence of PFOA (*n* = 68)

Independent variable	Dependent variable	Odds ratio (95% CI)*	
		Crude model	Propensity adjusted model
Calcaneus inverted angle, per degrees	No PFOA (*n* = 30) vs. ANY PFOA (*n* = 38)	**1.118 (1.018–1.245)** ^†^	**1.134 (1.013–1.291)** ^†^
	No PFOA (*n* = 30) vs. MIXED PFOA (*n* = 19)	**1.166 (1.031–1.356)** ^†^	1.135 (0.958–1.406)
	No PFOA (*n* = 30) vs. MEDIAL PFOA (*n* = 22)	**1.180 (1.044–1.368)** ^††^	**1.180 (1.005–1.439)** ^†^
	No PFOA (*n* = 30) vs. LATERAL PFOA (*n* = 26)	**1.109 (1.010–1.235)** ^††^	1.078 (0.965–1.213)

PFOA: patellofemoral osteoarthritis; 95% CI: 95% confidence interval

*Adjusted for propensity to prescribe as a function of age, (continuous), sex (0: male, 1: female), body mass index (continuous), tibiofemoral joint Kellgren/Lawrence grade (continuous), corrected anatomical axis angle (continuous), presence of varus thrust (0: absence, 1: presence), and knee flexion range of motion (continuous)

^†^*p* <0.05; ^†^^†^*p* <0.01

## Discussion

This exploratory study showed that patients with coexisting PFOA and medial TFOA on average had an inverted calcaneus 3.1° greater than those with isolated medial TFOA after adjusting for covariates, although approximately 70% of patients in both groups had a normal range of calcaneus angle. Increased calcaneus inverted angle was significantly associated with higher odds of any and medial PFOA and likely to be associated with higher odds of the presence of mixed and lateral PFOA. The association between rearfoot alignment was in the same direction for medial or lateral PFOA, thereby rearfoot alignment appears not to be associated with compartmental distribution of PFOA. Potential risk factors associated with PFOA involve patellar alignment relative to trochlea; muscle weakness, such as in the quadriceps; and abnormal biomechanics [38]. While an extensive literature review found similarities in clinical symptoms, structure, and physical function between patients with PF pain and PFOA, none of the included studies examined rearfoot posture in patients with PFOA [18]. Thus, this study is the first to show that rearfoot posture may be a potential modifiable factor associated with PF joint disease.

Excessive rearfoot eversion is suggested to lead to tibial and femoral internal rotation, subsequently resulting in increased quadriceps angle and higher lateral PF cartilage stress [15] in accordance with “law of valgus” (i.e. varus alignment increases the medial PF force and valgus alignment increases the lateral PF force) [39]. If this biomechanical theory is correct, everted and inverted calcaneus are associated with lateral and medial PFOA, respectively. This theory is supported by the significant relationship between calcaneus inverted angle and the presence of medial PFOA shown by the findings. However, calcaneus inverted angle is likely associated with lateral PFOA given that the lower limit of 95% CI of OR is near 1.0. These findings do not clearly support a biomechanical link between inverted rearfoot alignment and medial compartment-specific PFOA. Thus, inverted calcaneus may represent a clinical feature of multicompartmental disease. Further studies examining the biomechanical link between rearfoot abnormality and PFOA are warranted to support the findings from this exploratory study.

We found that calcaneus inverted angle was associated with a higher prevalence of medial PFOA. Conflicting evidence linking foot eversion and PF exists, although excessive foot eversion and its relationship to PF pain have been discussed in literature. For example, Powers et al. found that patients with PF pain exhibited increased rearfoot inversion compared to those without PF pain in younger adults when examined using a goniometer [20]. The presence of both PF pain and altered foot posture can lead to this progression given that PF pain in younger adults is suggested to be a precursor to PFOA. However, due to the cross-sectional nature of this study, PFOA may develop first and altered rearfoot posture may be a consequence of PFOA. Bidirectional segmental relationship has been determined among foot, shank, thigh, and pelvis [40]; therefore, a prospective cohort study on the incidence of PFOA in patients with inverted rearfoot but without PFOA should be conducted. This is particularly important given that risk factors associated with the incidence and progression of PFOA have not been fully determined.

It should be noted that there was a large inter-individual variability of calcaneus inverted angles, although patients with coexisting PFOA had an inverted calcaneus 3.1° greater than those with isolated medial TFOA. Understanding these variabilities is important because interventions concerning foot orthoses targeting PF joint disease may lack clinical significance [19, 41] and evaluating individual rearfoot posture may facilitate pain reduction of foot orthoses. Sultive et al. found that increased inverted calcaneus during standing is a potential indicator of non-success in the treatment of foot orthoses for improving PF pain [42], indicating a substantial role for rearfoot posture on foot orthoses in targeting the PF joint.

The current study included patients with medial TFOA and compared rearfoot posture in patients with and without PFOA because mixed OA is common [5–7] and is likely to be more painful than those with isolated PFOA [43]. However, the observed relationship between varus thrust and the presence of PFOA in patients with medial knee OA may not be true for patients with isolated PFOA that was suggested as a precursor of mixed OA [44].

There are some limitations to be noted. First, the cross-sectional study design limits our ability to identify causality between inverted rearfoot posture and PFOA. Second, a foot scanning system was used for static measurements while standing. Evaluating dynamic rearfoot alignment through three-dimensional motion capture apparatus [45] may provide substantial information about the association between foot posture and PFOA with higher accuracy and reliability than static measure [46]. Furthermore, calcaneus inverted angle does not include the subtalar joint and may yield different values compared to traditional evaluation methods that use goniometers for evaluating rearfoot posture [47]. Nevertheless, this scanning system is advantageous because it has a high accuracy for measuring static foot posture [29] which can be clinically assessed in a short amount of time. Third, PFOA identification using radiographs is an important limitation. Radiographic assessment indirectly measures the cartilage and is less sensitive than MRI. This would lead to differences in the prevalence of coexisting PFOA. Specifically, patients with isolated TFOA may have cartilage damage in the PF joint without radiographic evidence of PFOA. Furthermore, radiographic views might be affected by knee position and the patellar alignment. Fourth, this study included subjects who did not undergo follow-up x-ray is an important limitation. Some of the individuals who did not have PFOA at baseline might have developed radiographic PFOA at follow-up without worsen of clinical symptoms, which might affect the relationship between rearfoot alignment and presence of PFOA. Finally, this study did not account for confounders of PFOA, such as the quadriceps muscle [48] and gait kinematics [48, 49]. These possible confounders need to be examined further using epidemiologic studies to elucidate the relationship between rearfoot posture and PFOA.

## Conclusions

This exploratory study found that patients with coexisting PFOA on average had a 3.1° more inverted calcaneus than those with isolated medial TFOA after adjustment for covariates. Increased calcaneus inverted angle was significantly associated with higher odds of the presence of any and medial PFOA, and likely to be associated with higher odds of the presence of mixed lateral PFOA. Further studies are warranted to elucidate the pathomechanics linking rearfoot and PF joint disease.

## Abbreviations

AAA: Anatomical axis angle
BMI: Body mass index
ICC: Intra-class correlation coefficient
K/L grade: Kellgren/Lawrence grade
OA: Osteoarthritis
OR: Odds ratio
PF: Patellofemoral
PFOA: Patellofemoral osteoarthritis
ROM: Range of motion
SD: Standard deviation
TFOA: Tibiofemoral osteoarthritis
